# *Eriogynapyretorum* (Lepidoptera, Saturniidae) parasitoid species investigated in Fujian, China

**DOI:** 10.3897/BDJ.11.e108794

**Published:** 2023-08-30

**Authors:** Hao Yu Lin, Jia Jin Wang, Xin Jie Mao, Song Kai Liao, Hui Chen

**Affiliations:** 1 College of Forestry and Landscape Architecture, South China Agricultural University, Guangzhou, China College of Forestry and Landscape Architecture, South China Agricultural University Guangzhou China

**Keywords:** *Camphoraofficinarum*, biocontrol, parasite, Encyrtidae, Trichogrammatidae, Eupelmidae, Scelionidae, Ichneumonidae, Chalalcididae

## Abstract

*Eriogynapyretorum* Westwood is a notorious defoliator of *Camphoraofficinarum* Nees that causes large economic and ecological losses in planted forests. To understand the importance of suppressing the population of *E.pyretorum* on natural parasitoids, a four-years investigation was conducted in the field. Four egg parasitoid species *Ooencyrtuskuvanae* Howard, *Trichogrammachionis* Ishii, *Telenomus* sp. and *Anastatusdexingensis* Sheng & Wang were captured in the wild. One of these is the dominant endoparasitoid species *T.chionis*, which has a quicker developmental time (8.33 d), more offspring (8.39/egg) and a greater parasitism rate (89.54%). With different elevation distributions, the parasitism rates for *Kriechbaumerellalongiscutellaris* Qian & He, *Gregopimplahimalayensis* (Cameron), *Theroniadepressa* (Gupta) and *Xanthopimplakonowi* (Krieger) were 17.29%, 2.10%, 4.23% and 0.83%, respectively. Female longevity (47.75 d), offspring (13.36/pupa) and sex ratio (1.16:1) were compared in four pupal parasitoids and *K.longiscutellaris* was the most abundant species of *E.pyretorum* in Fujian Province.

## Introduction

*Camphoraofficinarum* Nees (Laurales, Lauraceae), whose variant name is *Cinnamomumcamphora* (L.) J. Presl, is widely cultivated in south China as an ornamental avenue tree because it has a good shape, rapid growth and can purify air, reduce air and noise pollution and maintain soil and water (Xiang et al. 2020). In addition, crude camphor extract from branches, leaves and roots of *C.officinarum* can be used as an insecticide or anticorrosive material (Yakefu et al. 2018, Tian et al. 2021). However, global climate change and rapid urbanisation have facilitated the spread of pests, resulting in serious challenges to the biological security of China (Xiang et al. 2020).

Currently, several defoliators are damaging *C.officinarum*, including *Pagiophloeustsushimanus* (Chen et al. 2020, Samartsev et al. 2021) and *Eriogynapyretorum* (Yin et al. 2008). Amongst these pests, *E.pyretorum* causes serious damage to *C.officinarum*, primarily by larvae feeding on leaves, which decreases photosynthesis and seriously affects normal tree growth and development (Zhou et al. 2021). Beta-cypermethrin and other insecticides can achieve 91.3% control of *E.pyretorum* larvae (Yin et al. 2008). However, chemical insecticides can negatively affect the environment by causing death of natural enemies and resistance in pests and leaving insecticide residues (Naqqash et al. 2016), so it is very important to find natural enemies that can control *E.pyretorum*. Research on natural enemies of *E.pyretorum* remains limited and little is known about the species and their biological characteristics (Yin et al. 2008, Zhou et al. 2021). Therefore, *E.pyretorum* were collected in the field and then reared in the lab to obtain natural parasitoid enemies. Biological characteristics of these parasitoids were examined in order to preliminarily assess its potential for release as a biological control agent (Holthouse et al. 2020).

## Material and methods

**Figure 1.**
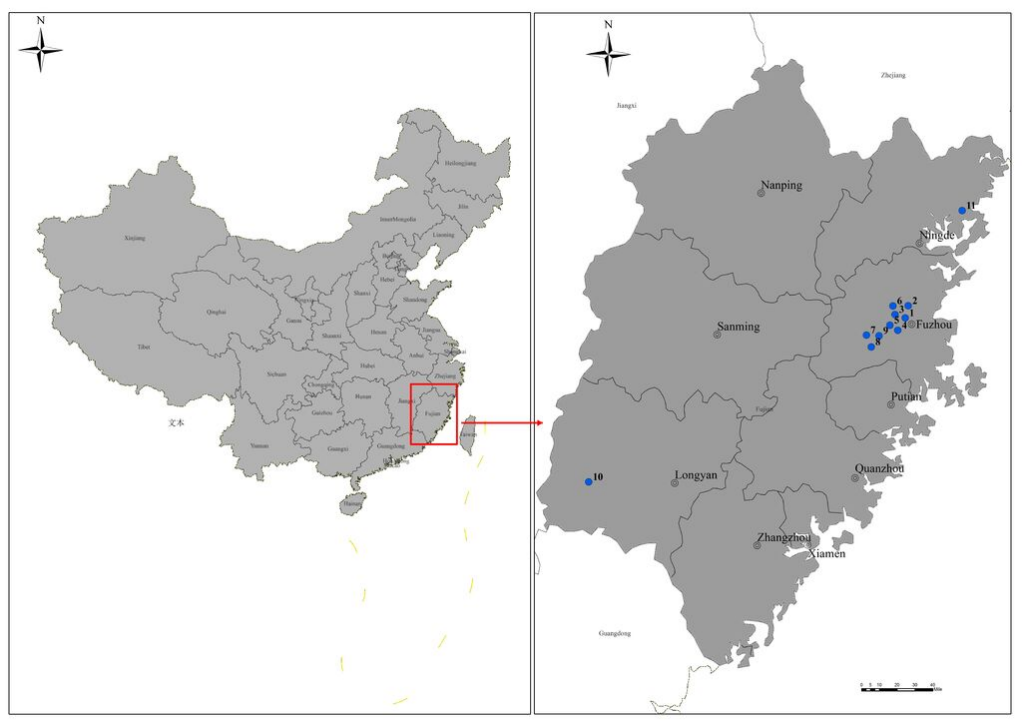
From 2019 to 2022, blue dots in the map represent the locations where masses of wild larvae and pupae will be released and collected. At Sites 1 and 11, *E.pyretorum* was identified. The following geographic coordinates are given: Fujian Agriculture and Forestry University is known as FAU (NO.1), Jinniushan Park is known as JNP (NO.2), Houmei Village is known as HMV (NO.3), Xiyuangong Road is known as XYR (NO.4), Xiyuan Village is known as XYV (NO.5), Guanzhong Village is known as GZV (NO.6), Chenjia Village is known as CJV (NO.7), Baidou Village is known as BDV (NO.8), Dangyun Village is known as DYV (NO.9), Yuanfu Village is known as YFV (NO.10) and Yangmei Ridge is known as XPR (NO.11).

In Fujian Province, 11 sample locations were found and looked into in accordance with the distribution of *C.officinarum* forest and its infestation by *E.pyretorum* (Fig. 1). Fujian Agriculture and Forestry University, (26°5′3″N, 119°14′13″E, ca. 110 m a.s.l., FAU); Jinniushan Park, Gulou District (26°5′6″N, 119°15′45″E, ca. 100 m a.s.l., JNP); Houmei Village, Minhou County (26°5′52″N, 119°11′58″E, ca. 40 m a.s.l., HMV); Xiyuangong Road, Minhou County (26°3′3″N, 119°10′42″E, ca. 30 m a.s.l., XYR); Xiyuan Village, Minhou County (26°3′36″N, 119°7′36″E, ca. 30 m a.s.l., XYV); Guanzhong Village, Minhou County (26°12′27″N, 119°10′59″E, ca. 50 a.s.l., GZV); Chenjia Village, Yongtai County (26°0′54″N, 118°54′20″E, ca. 600 a.s.l., CJV); Baidou Village, Yongtai County (25°54′55″N, 118°55′47″E, ca. 80 a.s.l., BDV); Dangyun Village, Yongtai County (25°59′32″N, 119°0′53″E, ca. 670 a.s.l., DYV); Yuanfu Village, Wuping County (25°12′49.62″N, 116°17′13.28″E, ca. 490 a.s.l., YFV); Yangmei Ridge, Xiapu County (26°51′22.11″N, 119°56′45.15″E, ca. 500 a.s.l., XPR). The vector shape file of the map from Resource and Environment Science and Data Center in China (https://www.resdc.cn/DOI/DOI.aspx?DOIID=122).

After a significant pest outbreak, *E.pyretorum* indoor-reared eggs were also hung in the *C.officinarum* forest with an egg parasitoid collection device from December to March of the following year and the device was collected 30 days later. Larvae and pupae of *E.pyretorum* were collected in Fujian Province, China, from January 2019 to December 2022; geographic and vegetation information are presented in Fig. 1. Larvae were reared at 25 ± 1°C and 50% ± 10% relative humidity (RH) on a daily supply of fresh leaves of *C.officinarum* in rearing boxes (18 × 11 × 6 cm) until pupation. Then, samples were placed in an insectary to collect parasitoids (Lin et al. 2023).

The ratio of parasitoids to hosts was 1:1 to produce more offspring with normal individuals. The inoculation conditions were 25 ± 1°C and 50% ± 10% RH for 24 hours, each with 30 to 60 eggs with three replicates.

After emergence, parasitoids were fed with 30% honey solution in an artificial climate chamber (MGC-300H, Shanghai Yiheng Co., Ltd., Shanghai, China) at 25 ± 1°C and 50% ± 10% RH. Adult parasitoids and exit holes were photographed with an SLR camera. Specimens were stored in 80% alcohol and then preliminarily identified according to Qian et al. (1987), He (2004) and Yang and Chen (2018). Further the specimens were sent to the taxonomists along with literature consultation to obtain the final species results (Krieger 1899, Ryu and Hirashima 1985, Gupta and Saxena 1987, Huang and Noyes 1994, Lin 1994, Zhang et al. 2005, Peng et al. 2020).

During the rearing process (24 ± 1°C and 60% ± 10% RH), after adults emerged, events such as the time of adults leaving from exit holes, male courtship, adults mating and female ovipositing into the host were recorded. In addition, oviposition behaviour was observed and the oviposition stages recorded. The number of offspring was recorded, distinguishing between male and female by the absence or presence of an ovipositor, number of offspring and longevity of parasitoid wasps; longevity was defined as the time period from emergence of parasitoids to death. Parasitism rate, offspring and adult longevity were calculated as the mean ± standard deviation. These data were analysed by IBM SPSS statistics 23 and subjected to one-way ANOVA analysis.

Parasitism rate (%) = parasitoids emerging from the host/number of hosts (pupae or eggs) * 100.

Offspring = number of parasitoid from hosts/number of hosts that emerged from this parasitoid.

## Results

We found that *Ooencyrtuskuvanae* Howard (Hymenoptera, Encyrtidae), *Trichogrammachionis* Ishii (Hymenoptera, Trichogrammatidae), *Telenomus* sp. (Hymenoptera, Scelionidae) and *Anastatusdexingensis* Sheng & Wang (Hymenoptera, Eupelmidae) attacked the eggs of *E.pyretorum* (Fig. 2). *Trichogrammachionis*, *Ooencyrtuskuvanae* and *A.dexingensis* had high parasitism rates of 89.54%, 87.78% and 85.56% in the lab, respectively, whereas *T.chilonis* had large numbers of offspring at 8.39 per egg; *A.dexingensis* had long longevity at 35.38 d; *T.chilonis* had a short development duration at 8.33 d (Table 1). Based on its higher parasitism rate (89.54%) and offspring (8.39/egg), *T.chionis* was determined to be the dominant egg parasitoid species. This species may have a synergistic effect on the suppression of the egg of *E.pyretorum* and be a promising candidate for widespread release to control caterpillars in *C.officinarum* forests.

In total, 827 *E.pyretorum* (56 larvae and 771 pupae) were collected in the field, of which 151 samples were parasitised (Fig. 3). Four species of parasitoids from two families were identified: *Gregopimplahimalayensis* (Cameron) (Hymenoptera, Ichneumonidae), where the larva is the parasitised host stage and the pupa is the parasitoid emerging stage (Fig. 4); *Theroniadepressa* (Gupta) (Hymenoptera, Ichneumonidae) (Fig. 5), *Xanthopimplakonowi* (Krieger) (Hymenoptera, Ichneumonidae) (Fig. 6) and *Kriechbaumerellalongiscutellaris* Qian & He (Hymenoptera: Chalcididae) (Fig. 7) are pupal parasitoids. *Gregopimplahimalayensis* and *T.depressa* were new parasitoids recorded within *E.pyretorum*.

The wasp *G.himalayensis* parasitised *E.pyretorum* larvae and emerged in its pupal stage, with 9.33 offspring emerging per pupa. *Xanthopimplakonowi* oviposited within the pupae of *E.pyretorum*. The wasp *T.depressa* parasite percentage from *E.pyretorum* was 4.23% and offspring was 1.25 per pupa. The parasitism rate of *K.longiscutellaris* was 17.29% and the average number of offspring within *E.pyretorum* was 13.36. Thus, of the four species obtained, *K.longiscutellaris* had the highest parasitism rate, longest longevity and highest number of offspring per host (Table 2). Additional research revealed that multiparasitism between *K.longiscutellaris* and *T.depressa* naturally occurs.

**Figure 8.**
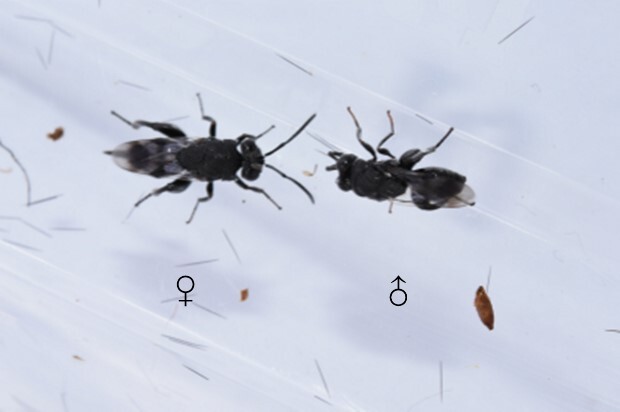
Courtship behaviour of *K.longiscutellaris*.

Emergence of *K.longiscutellaris* resulted in an average of seven exit holes per pupa, with diameters ranging from 2.51 to 4.75 mm. After emergence, adults could fly and forage within 2.55 ± 1.19 min. Male wasps surrounded females in courtship until a female received a male, which typically required 16.70 ± 4.30 min, but a few required 40 min (Fig. 8). Successful males pair-bonding and mated with females, with mating occurring within 6.85 ± 2.32 min.

Oviposition behaviour of *K.longiscutellaris* could be divided into three stages. In the search stage, female antennae drooped and the abdomen wiggled. In the investigation stage, after selecting a host, females extended the ovipositor to explore the best position for oviposition. If the host pupa wriggled in the cocoon, the female terminated the investigation and searched for the next position. In the spawning or oviposition stage, females inserted the ovipositor into the host gradually until the abdomen was close to the surface of the cocoon; this stage continued for 24.6 ± 4.78 min. With *E.pyretorum*, wasps only parasitised hosts within a cocoon shell.

## Discussion

According to Fang and Lian (1980), Li et al. (1986) and Qian et al. (1987), insects parasitising *E.pyretorum* include ten species from nine genera, six families and two orders. *Mesocomysalbitarsis* (Ashmead) and a *Trichogramma* sp. are egg parasitoids and an *Apanteles* sp. parasitises larvae. Six parasitoid wasps attack pupae, including *Habronyxpyretorum* (Cameron), *X.konowi* (Krieger), *Xanthopimplapedator* Fabricius, *Theroniazebradiluta* Gupta, *K.longiscutellaris* and *Brachymeria* sp. In addition, the parasitic fly *Exoristasorbillans* Wiedemann attacks *E.pyretorum* larvae (Lian and Fang 1980, Qian et al. 1987). In this work, we identified four parasitoid wasps within pupae of *E.pyretorum*, of which *T.depressa* and *G.himalayensis* were discovered as the first reported parasitoids of *E.pyretorum* in Fujian Province of China.

*Gregopimplahimalayensis* is widespread in North Korea, Japan and India and has been recorded in 14 provinces of China (Yang and Chen 2018). This parasitoid has multiple host species, including *Philosamiacynthia* Walker et Felder, *Dendrolimuspunctatus* Walker and *D.spectabilis* Butler (Yang and Chen 2018). *Theroniadepressa* was present in the Philippines and three provinces of China (Lin et al. 2017). The parasitoid has multiple host species, including *Artonafuneralis* (Butler) and *Dendrolimushoui* Lajonquiere. *Xanthopimplakonowi* was also distributed in Asia, including in Japan, Myanmar, Vietnam, India, Thailand, Malaysia and Indonesia. It has also been recorded in 13 provinces of China (Lian and Fang 1980). Lian and Fang (1980) and He (2004) identified ten host species, including *Philosamiacynthia* Walker et Felder and *Antheraeapernyi* (Guerin-Meneville). *Kriechbaumerellalongiscutellaris* was first recorded in Zhejiang Province, China, by Qian et al. (1987). Then, it was successively collected from *E.pyretorum*, *P.cynthia*, *D.punctatus*, *D.houi* and *Ceruramenciana* in China (Qian et al. 1987, He 2004). *Xanthopimplakonowi* and *T.depressa* are present in south China and other southeast Asian countries (He 2004, Lin et al. 2017). Both *G.himalayensis* and *K.longiscutellaris* are mainly distributed in north China (Qian et al. 1987, Yang and Chen 2018). These results show that wasps have a large latitudinal distribution and strong adaptabilities to different climates in a wide latitudinal range.

Unlike parasitoids captured in the wild, exit holes can validate the parasitic nature of the host and enable the determination of the body size (Lin et al. 2017). Furthermore, the number of exit holes can corroborate the quantity of parasitoids emerging from the same host and superparasitism can lead to an increase in the number of these holes (Tunca et al. 2017, Liu 2019).

Most male parasitoid wasps take a dominant role in mating activities, displaying a series of different types of behaviour to attract females. The courtship behaviour of parasitoids typically includes chasing, antennal touching and drumming and attempted copulation (Ardeh et al. 2004, Liu and Mottern 2017). Other types of behaviour include the 'Dancing' of the male *Bathypletescurculionis* (Dowell and Horn 1978) and the 'Swaying' of male *B.lasus* and *B.intermedia*, characterised by antennae raised at a 45° angle, while the body sways from side to side (Simser and Coppel 1980).

Previous studies have observed that parasitoid wasps typically have shorter mating durations. For example, the mating duration of *Brachymerialasus* is only 8 s, while mating of *B.intermedia* lasts between 7 and 12 s (Simser and Coppel 1980). The average courtship and mating period for *Aphelinusmaculatus* is 5.5 s (Li et al. 2021), while the mating duration for *Campoletischlorideae* is relatively longer at 162 s (Tian et al. 2023). The copulation time for *K.longiscutellaris* is 6.85 min, similar to that of *Kriechbaumerelladendrolimi* (Sheng et Zhong). This may be due to variations across different genera. Extended mating durations may serve to increase the chances of successful fertilisation or, as in the case with *K.dendrolimi*, it may involve the secretion of substances to prevent subsequent males from mating with the female (Lin et al. 2017).

Most *K.longiscutellaris* emerged within a span of 1–5 days, but a few emerge on day 55. The two sets of offspring might be from different females. Further dissection of host pupae revealed that parasitoids developed irregularly. In addition, 30.15% of pupae contained more than one dead adult. Female parasitoids likely oviposit many eggs, but the nutrition provided by host pupae may not be sufficient to satisfy growth and development of all parasitoid wasps (DaSilva et al. 2016). *Kriechbaumerellalongiscutellaris* has long adult longevity and *E.pyretorum* has a relatively long pupal stage (240 days; Zhou et al. (2021)). Consequently, females have sufficient time to search for suitable hosts in the field. Moreover, *K.longiscutellaris* has a high female:male ratio, which increases the diffusion rate and parasitism efficiency (Wang et al. 2013, Nava-Ruiz et al. 2021). Therefore, *K.longiscutellaris* has good potential for biological control of *E.pyretorum*.

For smaller natural enemies, such as *Telenomus* sp. or *Trichogramma* sp., current practice involves non-destructive DNA extraction to amplify COI (Xia et al. 2019), with specimens being retained for morphological identification. This approach can facilitate quick identification of the family or genus to which they belong (Liu and Mottern 2017). Mallet and Willmott (Mallet and Willmott 2003) suggest that it is best to use multiple gene fragments to distinguish species with similar morphology. When it comes to DNA strip coding, depending just on one gene fragment would not be reliable enough. Therefore, morphological methods cannot be completely abandoned.

## Conclusions

Four egg parasitoid species *Ooencyrtuskuvanae*, *Trichogrammachionis*, *Telenomus* sp., *Anastatusdexingensis* and four pupal parasitoid species *Kriechbaumerellalongiscutellaris*, *Gregopimplahimalayensis*, *Theroniadepressa*, *Xanthopimplakonowi* were captured within *Eriogynapyretorum* in 11 sample locations of Fujian Province. *Trichogrammachionis and K.longiscutellaris* were the most dominant egg parasitoid and pupal parasitoid of *E.pyretorum*, respectively.

## Figures and Tables

**Figure 2a.**
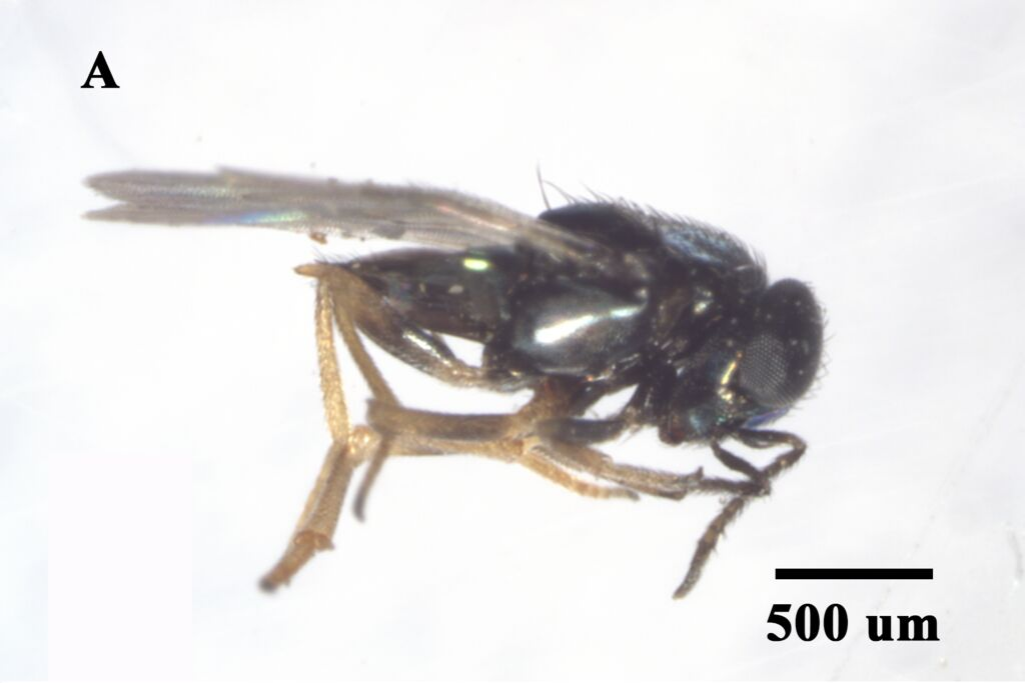
Adult of *Ooencyrtuskuvanae*;

**Figure 2b.**
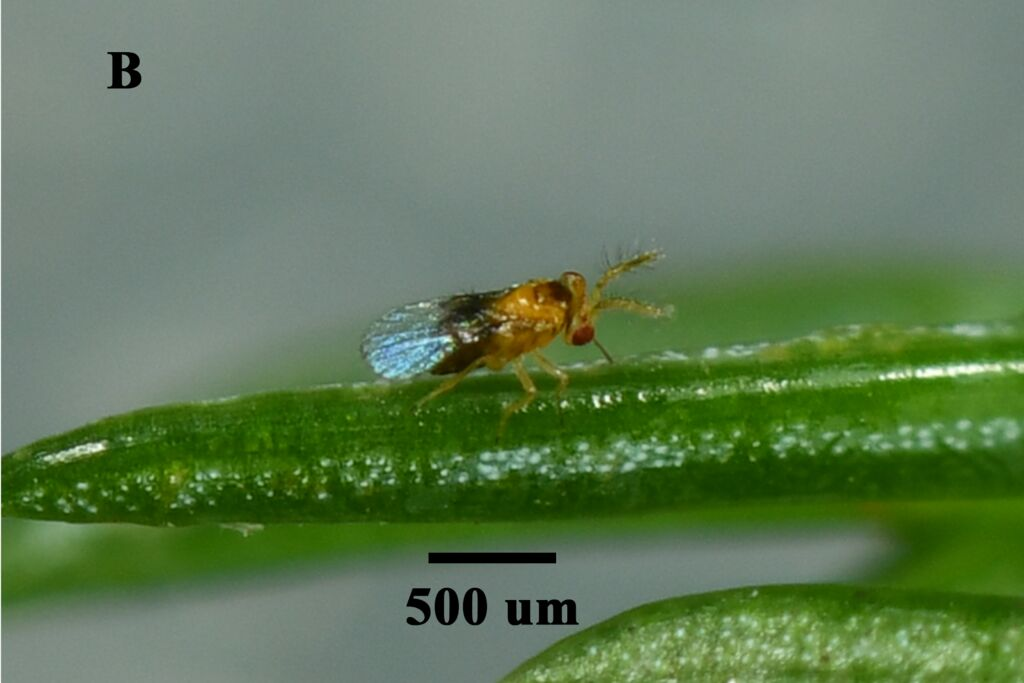
Male of *Trichogrammachionis*;

**Figure 2c.**
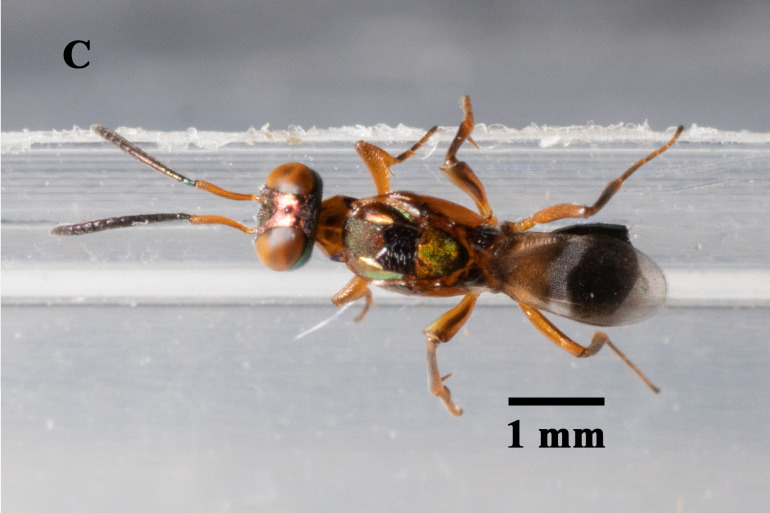
Adult of *Anastatusdexingensis*;

**Figure 2d.**
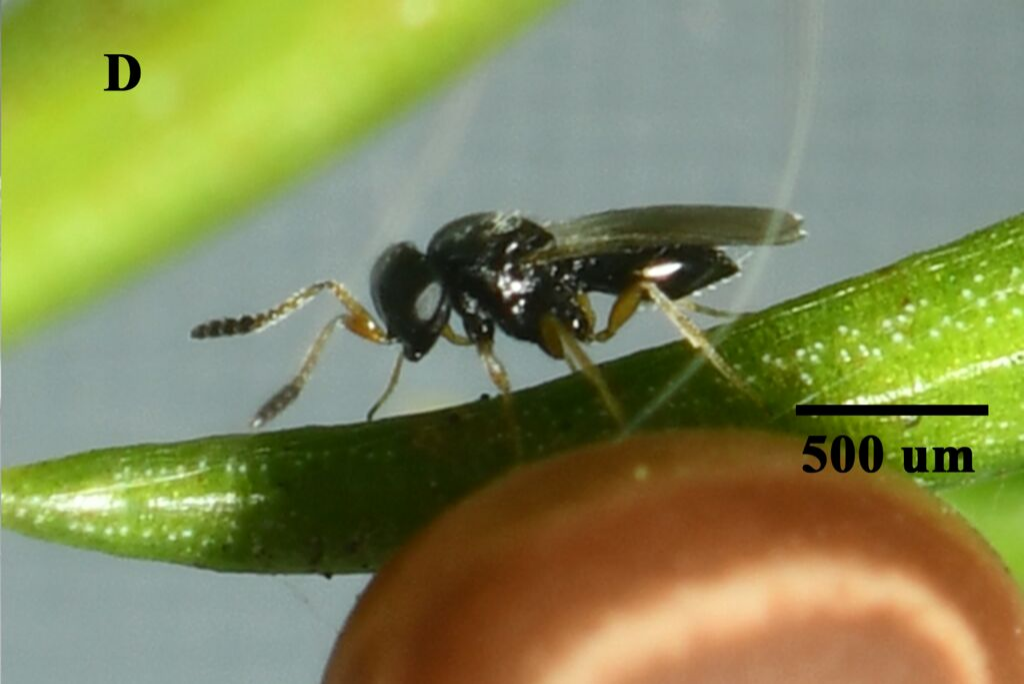
Adult of *Telenomus* sp.

**Figure 3a.**
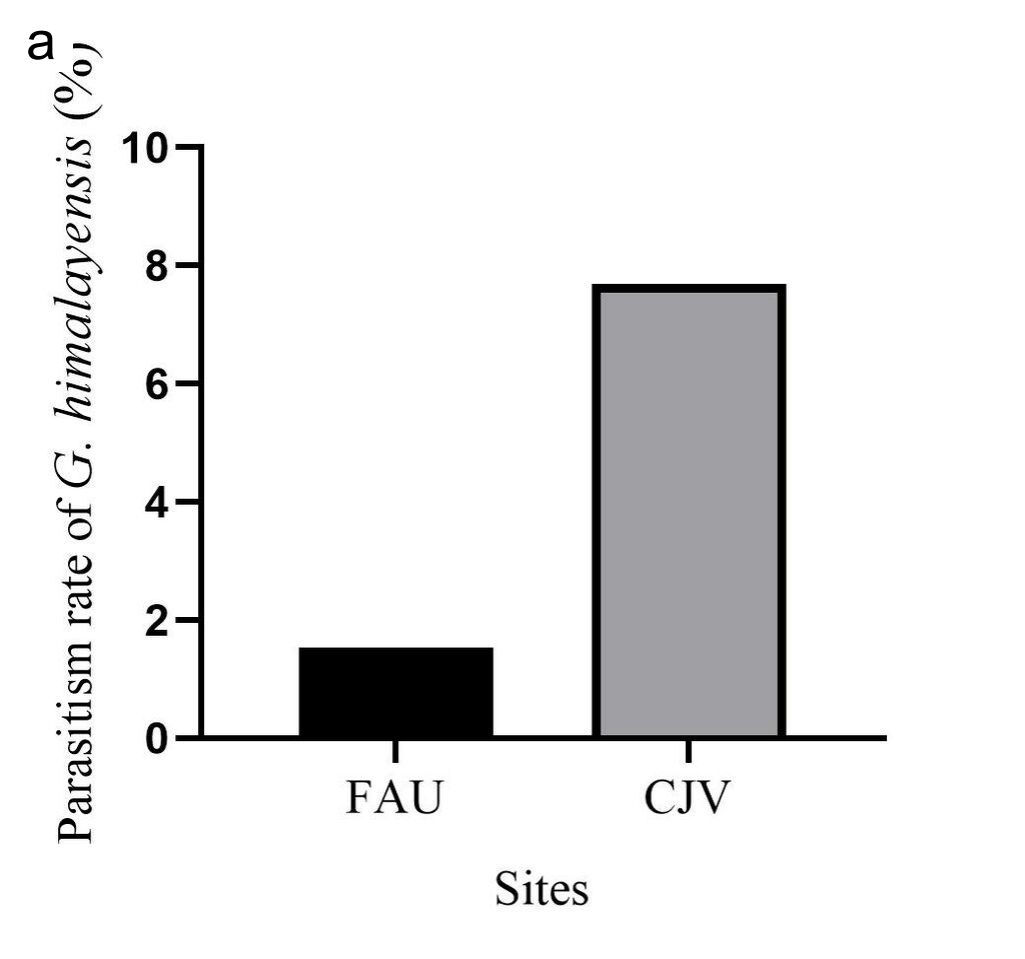


**Figure 3b.**
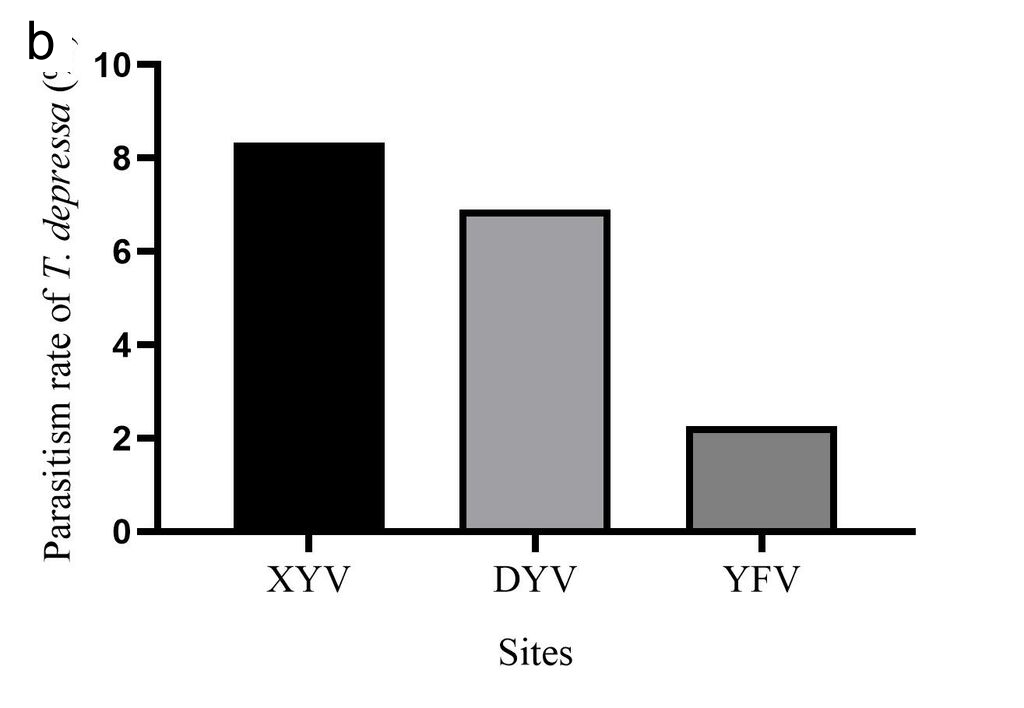


**Figure 3c.**
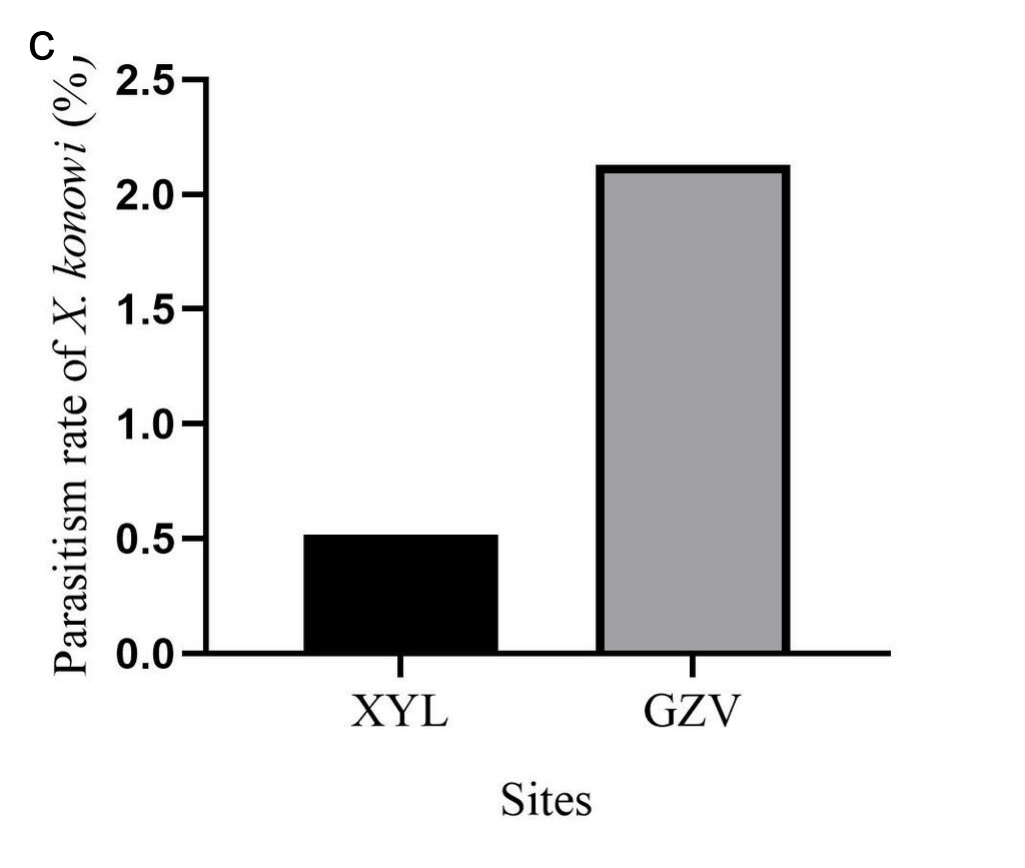


**Figure 3d.**
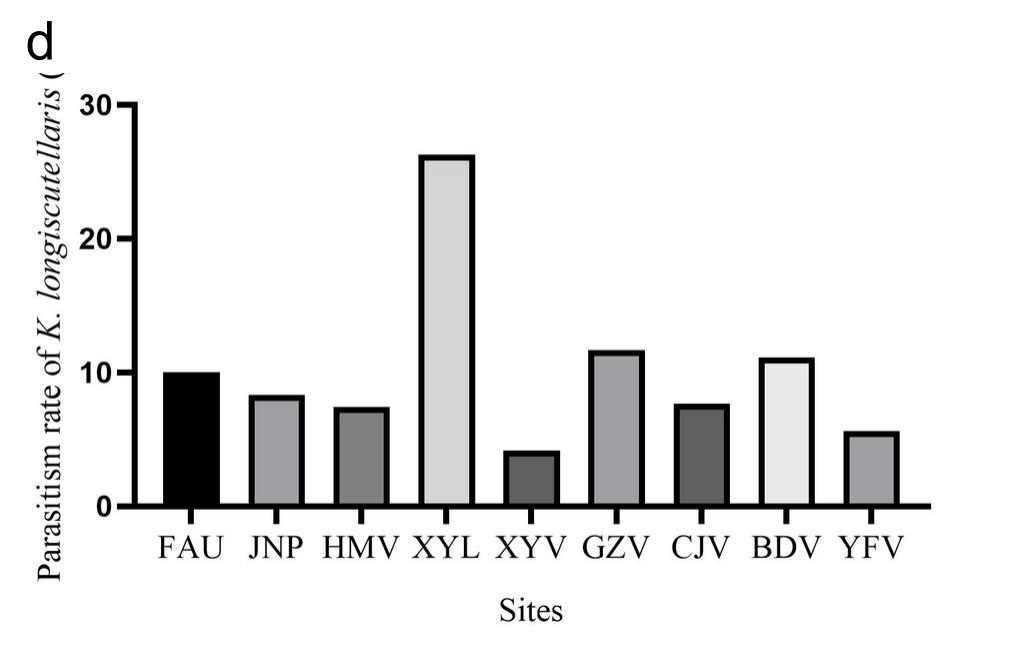


**Figure 4a.**
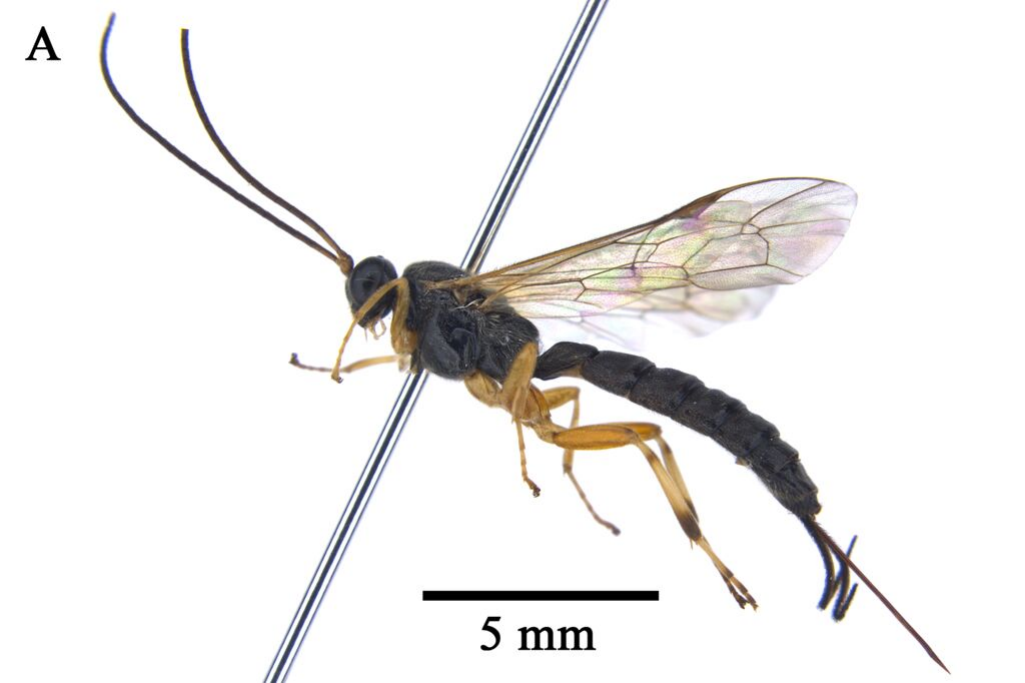
Female of *G.himalayensis*;

**Figure 4b.**
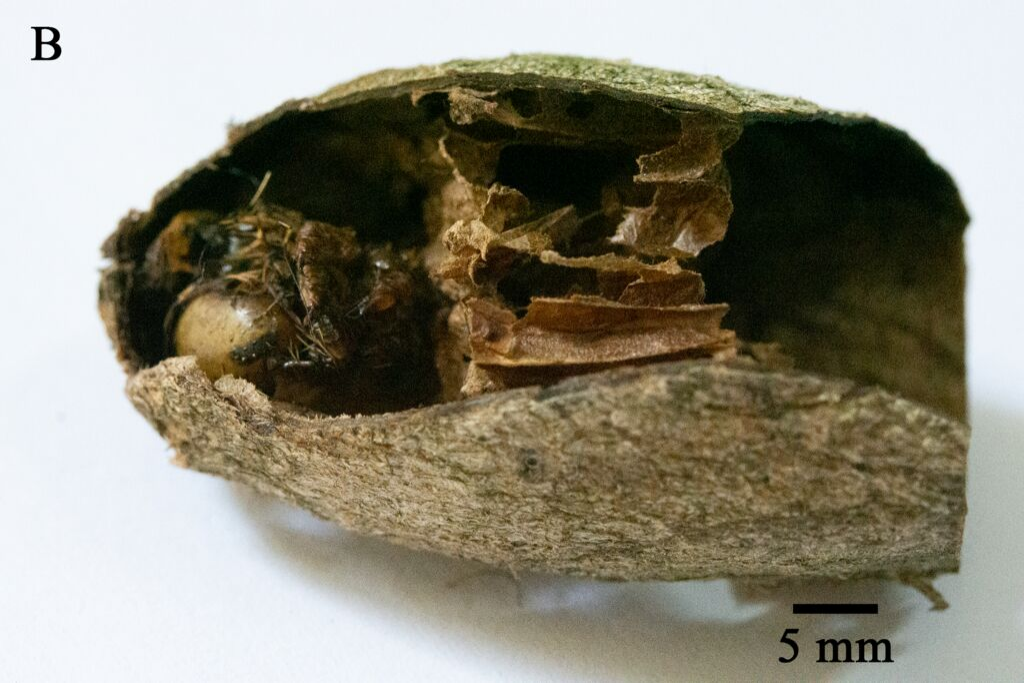
Exit hole of *G.himalayensis*.

**Figure 5a.**
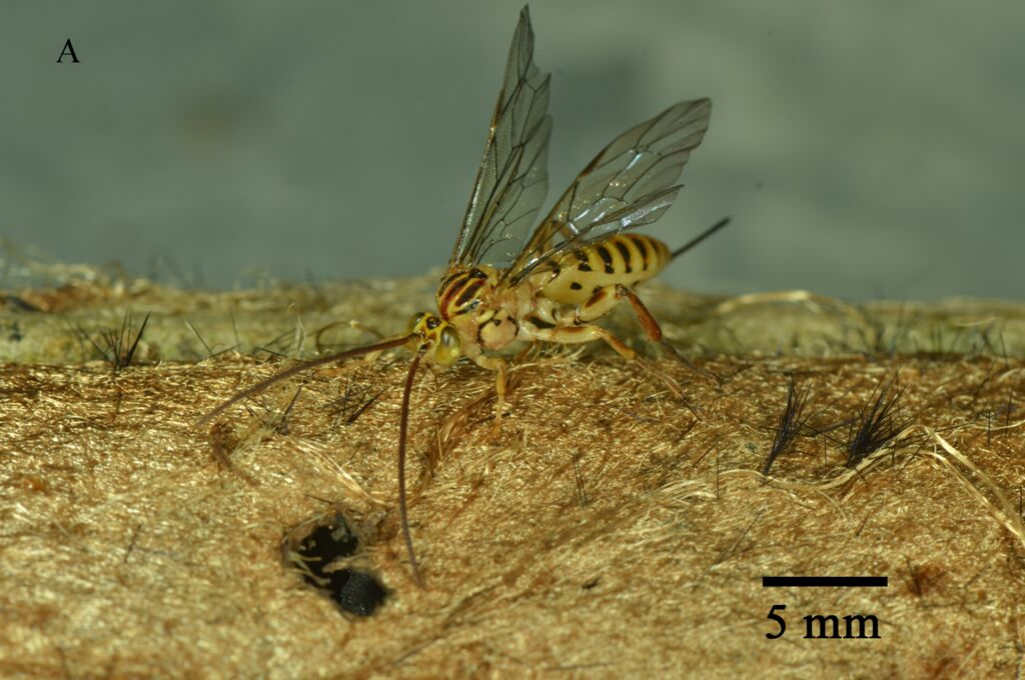
Female of *T.depressa*;

**Figure 5b.**
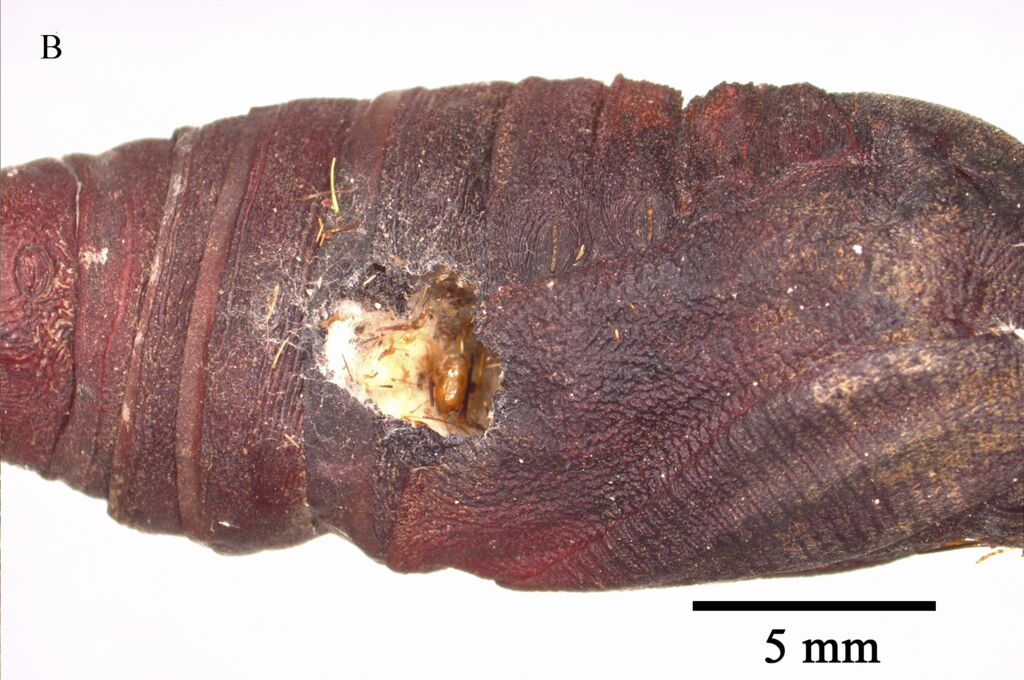
Exit hole of *T.depressa*.

**Figure 6a.**
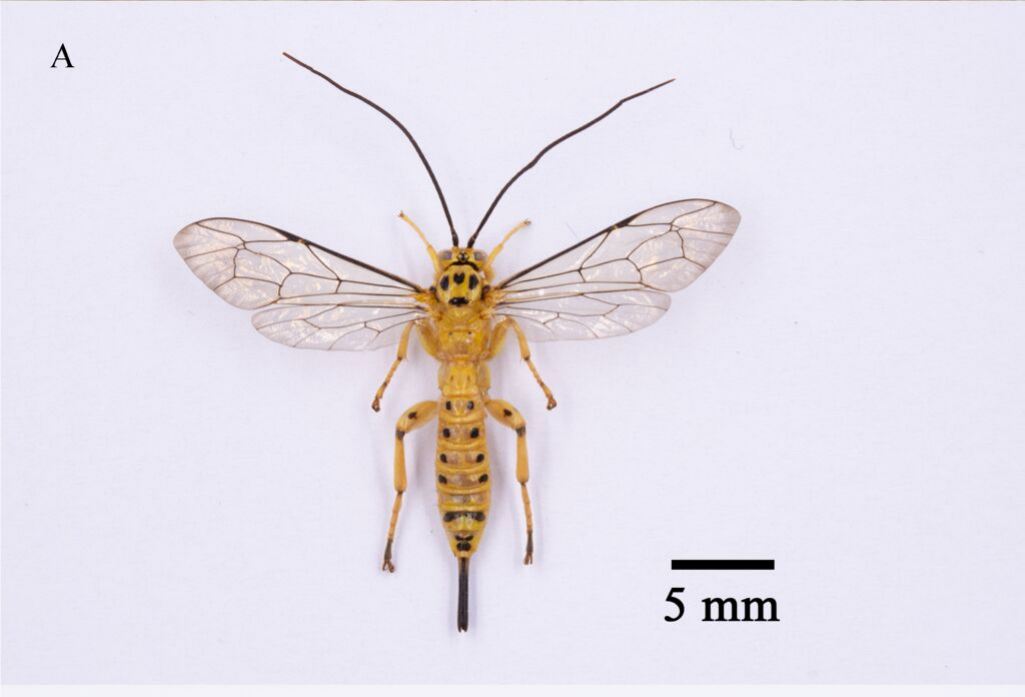
Female of *X.konowi*;

**Figure 6b.**
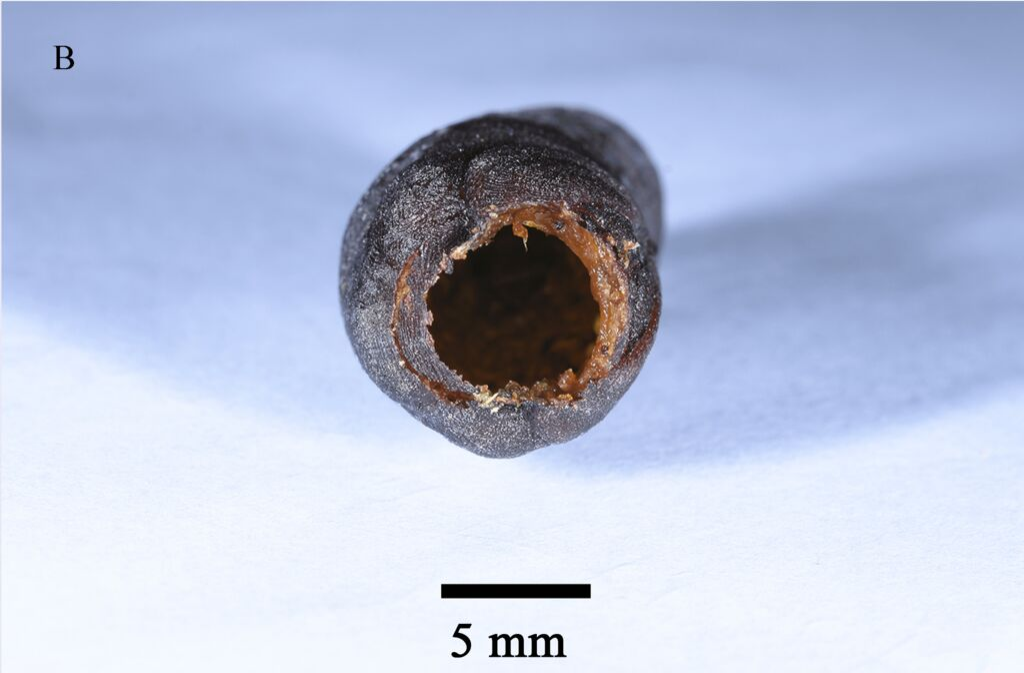
Exit hole of *X.konowi*.

**Figure 7a.**
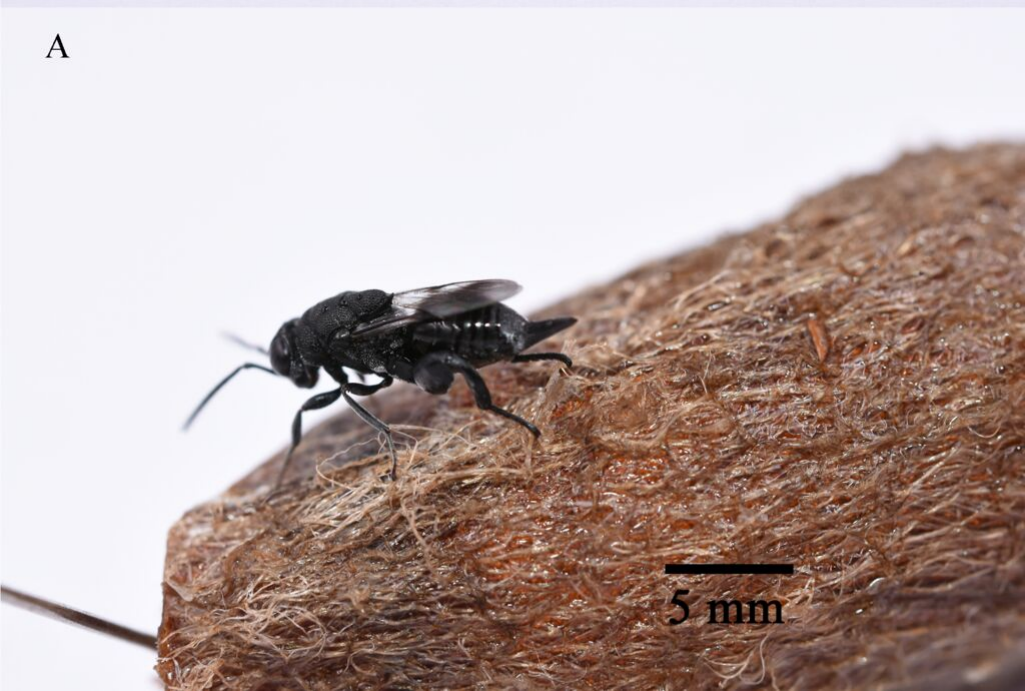
Female of *K.longiscutellaris*;

**Figure 7b.**
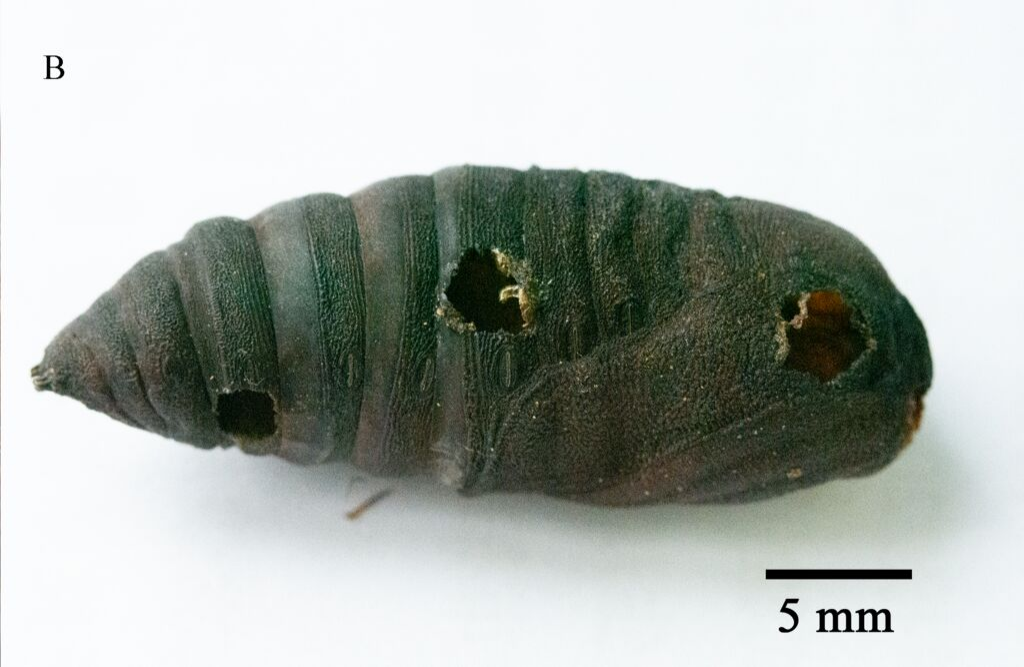
Exit holes of *K.longiscutellaris*.

**Table 1.** Biological characteristics of egg parasitoids in Fujian Province, China.

Species	Family	Parasitism rate (%)	Offspring	Ratio female: male	Longevity (d)	Developmental period (d)
*Ooencyrtuskuvanae*	Encyrtidae	87.78 ± 4.16 a	6.54 ± 0.52 b	1:0	7.35 ± 0.64 d	16.97 ± 0.34 b
*Trichogrammachionis*	Trichogrammatidae	89.54 ± 2.63 a	8.39 ± 0.60 a	2.79:1	9.97 ± 0.50 c	8.33 ± 0.47 c
*Telenomus* sp.	Scelionidae	21.73 ± 7.74 b	6.63 ± 1.09 b	1.67：1	25.98 ± 0.94 b	15.9 ± 0.25 b
*Anastatusdexingensis*	Eupelmidae	85.56 ± 6.14 a	1.13 ± 0.02 c	11.75：1	35.38 ± 0.23 a	44.91 ± 2.70 a

Note: Average number of offspring or longevity ± standard deviation.

**Table 2.** Biological characteristics of pupal parasitoids.

Species	Family	Parasitism rate (%)	Offspring	Ratio female: male	Female longevity (d)	Male longevity (d)
*Gregopimplahimalayensis*	Ichneumonidae	2.10	9.33	1.55: 1	9.1 ± 0.48	6 ± 0.76
*Theroniadepressa*	Ichneumonidae	4.23	1.25	1:0	13.80 ± 3.76	-
*Xanthopimplakonowi*	Ichneumonidae	0.83	1	3: 1	10.50 ± 0.50	7
*Kriechbaumerellalongiscutellaris*	Chalcididae	17.29	13.36	1.16: 1	47.75 ± 26.74	30.77 ± 14.20

Note: Average number of days of longevity ± standard deviation.
